# World Malaria Day 2021: Commemorating 15 Years of Contribution by the United States President’s Malaria Initiative

**DOI:** 10.4269/ajtmh.21-0432

**Published:** 2021-04-23

**Authors:** Richard W. Steketee, Misun Choi, Anne Linn, Lia Florey, Matthew Murphy, Rajesh Panjabi

**Affiliations:** 1 United States President’s Malaria Initiative, Centers for Disease Control and Prevention, Atlanta, Georgia;; 2 United States President’s Malaria Initiative, United States Agency for International Development, Washington, District of Columbia

## Abstract

World Malaria Day 2021 coincides with the 15th anniversary of the United States President’s Malaria Initiative (PMI) and follows the first anniversary of the declaration of the coronavirus disease (COVID-19) pandemic. From 2006 to the present, the PMI has led to considerable country-managed progress in malaria prevention, care, and treatment in 24 of the highest-burden countries in sub-Saharan Africa and three countries in the Southeast Asia Greater Mekong subregion. Furthermore, it has contributed to a 29% reduction in malaria cases and a 60% reduction in the death rates in sub-Saharan Africa. In this context of progress, substantial heterogeneity persists within and between countries, such that malaria control programs can seek subnational elimination in some populations but others still experience substantial malaria disease and death. During the COVID-19 pandemic, most malaria programs have shown resilience in delivering prevention campaigns, but many experienced important disruptions in their care and treatment of malaria illness. Confronting the COVID-19 pandemic and building on the progress against malaria will require fortitude, including strengthening the quality and ensuring the safety and resiliency of the existing programs, extending services to those currently not reached, and supporting the people and partners closest to those in need.

We are celebrating World Malaria Day 2021 in a changed world. This past year, the coronavirus disease (COVID-19) pandemic drastically changed our individual daily routines as well as our approach to the spectrum of infectious diseases, their prevention and control, and our protection. It is easy to forget that malaria is an age-old pandemic, but there are many parallels and overlaps (as well as some contrasts) between malaria and our new pandemic.

This year also marks the 15th anniversary of the United States President’s Malaria Initiative (PMI) and includes the 15th Annual Report to Congress^1^ describing the United States government’s contributions to decreasing malarial infection, illness, and death worldwide. The PMI was conceived in 2005 to scale-up access to newly available evidence-based malaria innovations, which have grown to include rapid diagnostic tests (RDTs), artemisinin combination treatments (ACTs), insecticide-treated bed nets (ITNs) and indoor residual spraying (IRS) with new and more effective insecticides, intermittent preventive treatment during pregnancy, and seasonal malaria chemoprevention (SMC). In 2006, the PMI began malaria control support for people at risk in malaria-endemic Angola, Tanzania, and Uganda, with an initial annual budget of US$30 million.^2^ This support coincided with increasing investments in malaria control by endemic countries, the Global Fund to Fight AIDS, Tuberculosis, and Malaria, the Bill & Melinda Gates Foundation, the World Bank, the Government of the United Kingdom, and other donors. Early successes in reducing malaria disease and death, bipartisan support from the United States Congress, and the generosity of the American people have allowed PMI to expand its support and geographic coverage. PMI now provides support to an estimated 725 million residents in 27 countries with the highest burden of malaria (24 in sub-Saharan Africa and 3 in the Southeast Asia Greater Mekong subregion); the majority of the annual malaria budget of the United States Agency for International Development (USAID), approximately US$770 million, is directed to the PMI’s work (Figure 1).

**Figure 1.**
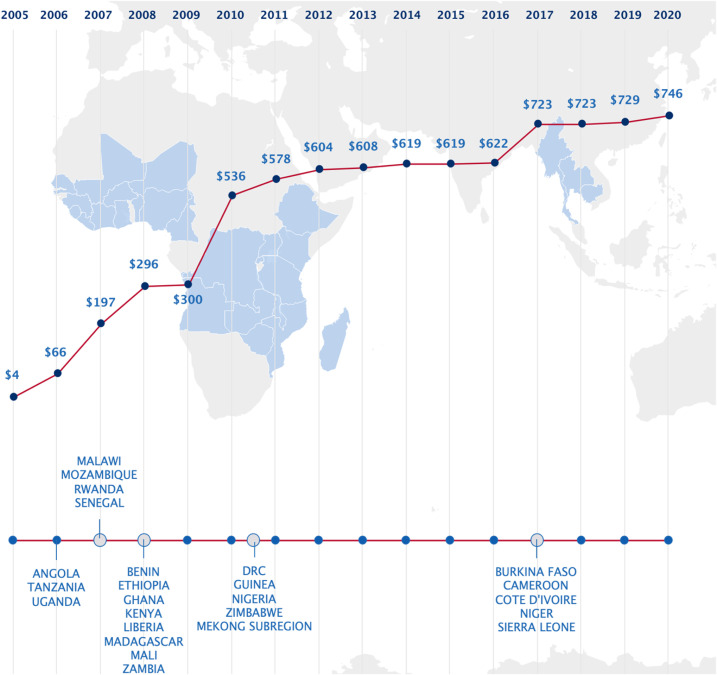
United States President’s Malaria Initiative (PMI) country engagement and financial investment (in millions of dollars) in malaria control over the course of 15 years. The bottom timeline notes PMI partner country expansion. The red line shows only PMI funding (in millions of dollars); however, the United States Agency for International Development (USAID) also funds malaria activities outside of PMI, including support to Burundi and the Latin American and the Caribbean regions, multilateral malaria efforts, and upstream research investments in malaria vaccines, drugs, and insecticide tools.

Although funding for malaria control from endemic countries and donors increased steadily from the early 2000s, since 2014, global malaria funding has plateaued at approximately US$3 billion per year (based on the 2019 dollar standard in the United States).^3^ Since the early 2000s, steady population growth in malaria-endemic countries has resulted in a substantially larger population at risk for malaria. This scenario equates to a decline in available per-capita resources and threatens continued progress toward malaria reduction and elimination goals.

Between 2005 and 2019, PMI-supported African countries experienced an estimated 49% increase in their populations at risk for malaria (Figure 2). Despite this population growth, the coverage of proven interventions, including vector control, case diagnosis and treatment, and preventive use of antimalarial drugs, has increased because of the program support and greater efficiencies in delivering these interventions.^4,5^ Since 2005, sustained expansion of intervention coverage in these countries has proven beneficial, with a 32% decrease in malaria case incidence and a 44% decrease in all-cause child mortality (ACCM), of which a substantial proportion is attributable to malaria (Figure 2).

**Figure 2.**
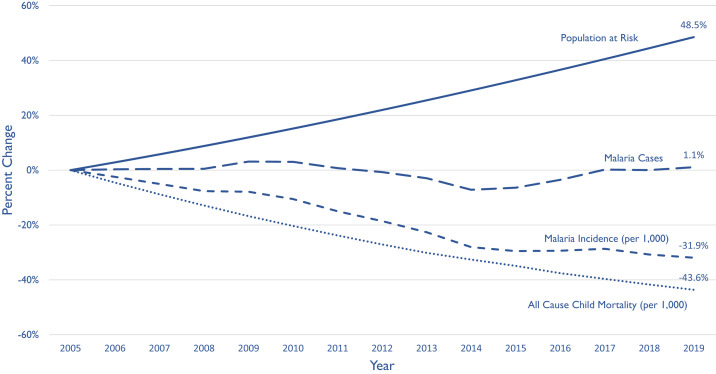
Trends in population growth, malaria cases, malaria case incidence, and all-cause child mortality (ACCM) in 24 PMI-focused sub-Saharan African countries from 2005 through 2019 (coinciding with PMI support). ACCM represents deaths from all causes among children younger than 5 years per 1000 live births (source: United Nations International Children's Emergency Fund [UNICEF] modeled data through 2019).^6^ Malaria cases represent the annual number of malaria cases (source: modeled data from the 2020 World Malaria Report).^7^ Malaria incidence represents the annual malaria cases per 1,000 population (source: modeled data from the 2020 World Malaria Report).^7^ Population represents the population at risk for malaria (source: modeled data from the 2020 World Malaria Report).^7^

National data from the PMI-engaged countries combined with modeling studies suggest that many of these countries experienced reductions in the *Plasmodium falciparum* malaria parasite prevalence rate (PfPR) in 2- to 10-year-old children. Only two of the countries had an ACCM less than 70 per 1,000 live births in 2005; however, as of 2019, half of the countries have achieved this level.^6^ Half of these countries had an estimated PfPR more than 20% in 2005, whereas only one-third had such a high prevalence in 2019^7^ (Figure 3A and B).

**Figure 3.**
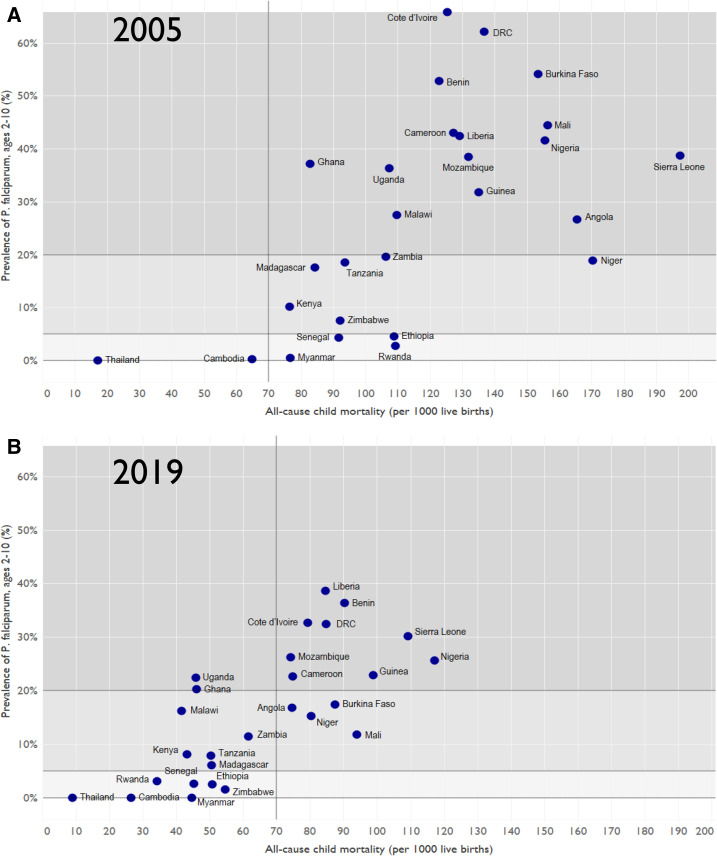
Reductions in malaria morbidity and mortality among 27 PMI-focused countries. National estimates of *Plasmodium falciparum* parasite prevalence rate (PfPR) and all-cause child mortality (ACCM) status in 2005 (**A**) and 2019 (**B**) are presented. ACCM represents deaths from all causes among children younger than 5 years per 1,000 live births (source: United Nations International Children's Emergency Fund [UNICEF] modeled data through 2019).^6^ Prevalence of *Plasmodium falciparum* (PfPR) malaria in children 2 to 10 years of age (source: modeled data from the Malaria Atlas Project).^7^ The ACCM categories (< 70 and ≥ 70 child deaths per 1,000 live births) and PfPR categories (< 5%, 5–20%, and > 20% annual national estimates) are not standard groupings; however, they are shown here for illustrative purposes.

The 2020 World Malaria Report^3^ from the World Health Organization (WHO) highlights the success of global malaria prevention efforts over the past two decades and also cautions about stalling progress. To increase momentum, the WHO has called for more attention to 10 large high-burden to high-impact (HBHI) countries in sub-Saharan Africa (Nigeria, Democratic Republic of the Congo, Mozambique, Uganda, Burkina Faso, Ghana, Niger, Cameroon, Mali, United Republic of Tanzania) and India, which together contribute more than two-thirds of the worldwide malaria cases.^8^ PMI supports all 10 HBHI countries in sub-Saharan Africa as well as 14 additional countries with smaller populations but a high malaria burden.

Despite overall reductions in ACCM and PfPR from 2005 to 2019 (Figure 3A and B), substantial heterogeneity in intervention coverage and health impacts across countries continues and underscores the importance of tailoring interventions to the unique circumstances of each country. For example, by 2019, modeled national estimates of ACCM in PMI-focus countries ranged from approximately 35 to 120 deaths of children younger than 5 years per 1000 live births, and the PfPR ranged from < 1% to nearly 40%. By grouping countries using summary estimates and illustrative cut-offs of parasite prevalence (< 5%, 5–20% and > 20%) and ACCM1(< 70 and > 70 deaths of children younger than 5 years per 1,000 live births) (Figure 3B), countries can be identified that share similar environmental, demographic, epidemiologic and health system characteristics that then influence malaria transmission, prevention, diagnosis, treatment and reporting. Analyses of the intervention impact and information exchange among countries with similar characteristics may help guide future program priorities. For example, countries with ACCM less than 70 and PfPR less than 5% (lower left corner of Figure 3B) may consider strategies to attain malaria elimination, whereas countries with higher ACCM and higher PfPR (upper right of Figure 3B) will need to reduce the rates of case incidence and case fatality before embarking on elimination.

Many countries have spatial heterogeneity in their rates of malaria transmission, illness, and death. By identifying and tracking this heterogeneity, countries can determine which districts might be poised for subnational elimination efforts and which districts should focus on transmission, illness, and death reduction before setting elimination goals. New approaches to data collection and analysis have helped countries to assess and track malaria disease and program heterogeneity, including time-specific geo-located data regarding morbidity, mortality, intervention coverage, supply stocks, and health service capacity (facilities, community posts, and community health workers), and to aligning malaria control strategies to the specific needs and capacities of each area.^9^ PMI is now investing in key actions to strengthen the capacity and reach of information systems, ensure timely supply and transport of preventive and curative commodities, foster partnerships to support local action, and extend life-saving interventions to the most difficult to reach and highest-risk populations, including migrants, displaced individuals, and those living in rural and remote areas.

The COVID-19 pandemic has threatened this steady 15 years of progress in controlling malaria.^10^ Soon after the WHO declared the COVID-19 pandemic, the PMI staff worked with malaria teams and the global community to adapt malaria technical guidance in response to the pandemic.^11^ Despite numerous challenges during 2020, among the PMI-supported sub-Saharan African countries, nearly all 34 previously planned intervention campaigns in 19 countries were completed with the targeted coverage and minimal delays, including distributing ITNs (all 13 planned countries), distributing SMC (all 9 planned countries), or providing IRS (11 of 12 planned countries). The standard practices of in-country advanced planning and management (e.g., supplying, training, deploying, and logistics management) and global collaboration to adapt these campaigns were crucial to this success.

Adapting standard case management of fever and malaria illness in health facilities and communities has proven to be more challenging during the COVID-19 pandemic. For example, early messages about COVID-19 prevention advised sick or febrile people to stay home unless they were severely ill. These messages conflicted with long-standing malaria guidance to seek health care early if a fever develops or if malaria is suspected. Before the COVID-19 pandemic, handwashing and the use of gloves were commonplace, but many healthcare workers did not routinely use masks, hand sanitizer, or other personal protective equipment (PPE) during patient care. The surge in demand for PPE, the challenges of procurement and transport for these products and other health commodities (especially during local or national lockdowns), and the early messages to stay home led to distrust and fear of healthcare systems and marked reductions in the seeking and provision of care. As the impact of COVID-19 transmission, evolving vaccination planning, and other infection prevention and control strategies ensue, countries with a high malaria burden are faced with the daunting task of preventing and managing two common causes of potentially fatal febrile illness with similar nonspecific signs and symptoms. Fortunately, the widespread use of rapid point-of-care malaria tests should provide for faster adoption of rapid tests for severe acute respiratory syndrome coronavirus 2 (SARS-CoV-2) antigen and hasten diagnosis, triage, and appropriate clinical care.

Challenges in adapting routine malaria case management services during the COVID-19 pandemic prompted alarming predictions that malaria cases and deaths would increase as COVID-19 spread.^10^ This concern was not unfounded; during the 2014 to 2016 Ebola virus epidemic in western Africa, cases of malaria and other non-Ebola diseases increased as people deferred medical care and the limited number of healthcare workers, commodities, and facilities were redirected to Ebola response activities.^12^ There are indications that malaria testing and treatment have been significantly disrupted by the COVID-19 pandemic.^13^ Primary healthcare has undergone pressure and community healthcare workers who deliver malaria services may have limited their work because of the lack of PPE, may have become ill themselves, or may have been diverted to COVID-19 priority areas.^14^ Delays in malaria diagnosis and treatment can lead to increases in mortality. It is plausible that in some high-burden malaria countries, excess deaths from malaria as an impact of the COVID-19 pandemic could exceed deaths directly caused by SARS-CoV-2. To prevent COVID-19 from reversing the enormous progress against malaria, continued commitment and technical support will be essential. These investments should support strengthened PPE and vaccination delivery and include hazard pay for facility-based and community health workers who are essential to sustaining malaria case management.^14^

Despite recent setbacks in malaria case management because of the COVID-19 pandemic, optimism about the capacity of malaria-endemic countries to reduce the burden of malaria or eliminate the disease should not be diminished.^15^ This past year has revealed the remarkable resiliency of dozens of malaria control campaigns and the adaptability of case management services in many countries. This success has been fueled by broad support and the talent, conviction, creativity, and energy of country program staff, community leaders, and health workers at all levels. For malaria (and many other disease control programs), critical support from the global community remains essential amid the devastating COVID-19 pandemic and will demand strong prevention and service platforms that are well-supplied, laboratory-able, and data-informed, and that emphasize community delivery to expand reach to the most remote populations at risk. As the PMI celebrates its 15th anniversary, we thank the global malaria community, country leadership, and at-risk communities for their partnership in this fight against malaria.
